# Alfred Russel Wallace and the Antivaccination Movement in Victorian England

**DOI:** 10.3201/eid1604.090434

**Published:** 2010-04

**Authors:** Thomas P. Weber

**Affiliations:** Ispra, Italy

**Keywords:** Vaccination, Alfred Russel Wallace, vaccines, England, historical review

## Abstract

Alfred Russel Wallace, eminent naturalist and codiscoverer of the principle of natural selection, was a major participant in the antivaccination campaigns in late 19th-century England. Wallace combined social reformism and quantitative arguments to undermine the claims of provaccinationists and had a major impact on the debate. A brief account of Wallace’s background, his role in the campaign, and a summary of his quantitative arguments leads to the conclusion that it is unwarranted to portray Victorian antivaccination campaigners in general as irrational and antiscience. Public health policy can benefit from history, but the proper context of the evidence used should always be kept in mind.

## Abstract 2

Historical analysis can play a major role in public health policy.

In 2009, the scientific community commemorated the 200th birthday of Charles Darwin and the 150th anniversary of the publication of On the Origin of Species by Means of Natural Selection. These occasions also directed the view of a wider public to the unjustly neglected figure of Alfred Russel Wallace (1823–1913) (Figure), explorer and codiscoverer of the principle of natural selection. In the past few years, Wallace’s work has in fact enjoyed increasing attention among the historians of science, as several new biographies and studies prove (*1*–*5*). But unlike Darwin, Wallace always was and probably will remain a serious challenge to the history of science: he stubbornly refuses to fit into the mold of the typical scientific hero. Wallace made without any doubt lasting contributions to biologic science, but the second half of his life was by and large devoted to what from today’s perspective are utterly lost causes: He became a passionate advocate of spiritualism, supported land nationalization, and fervently objected to compulsory smallpox vaccination.

**Figure:**
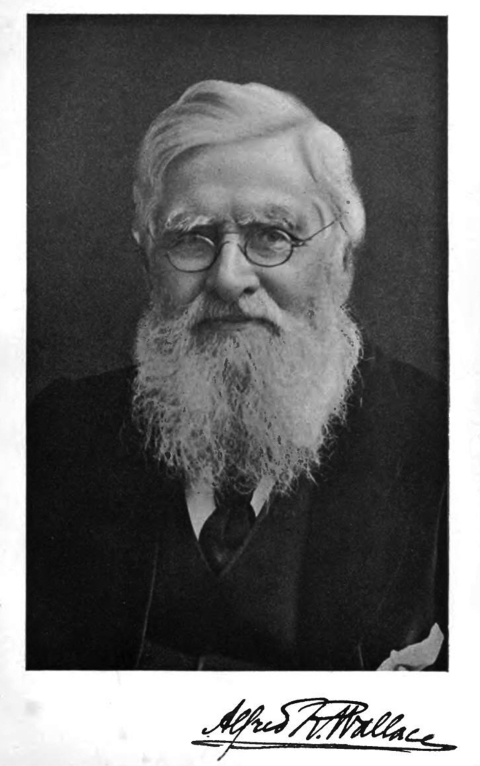
Alfred Russel Wallace (1823–1913). Perhaps best remembered today in history of science as the codiscoverer of the principle of natural selection, Wallace also played a prominent role in the antivaccination movement in late 19th century England.

The motives behind Wallace’s campaigns are sometimes difficult to fathom. He published copiously because this served for a long time as his major source of income, but these writings only show the public face of Wallace. Unlike Darwin, Wallace did not leave behind a large number of private letters and other personal documents; therefore, his more private thoughts, motives, and deliberations will probably remain unknown.

I provide a short introduction to Wallace’s life and work and then describe his contributions to the British antivaccination campaigns. Wallace’s interventions were influential; he was popular and well liked inside and outside scientific circles and, despite his controversial social reformism, commanded deep respect for his achievements and his personal qualities until the end of his long life.

I also briefly analyze the similarities and differences between the Victorian and contemporary vaccination debates. It has recently been argued that comparative historical analysis can play a major role in public health policy (*6*,*7*). In contemporary vaccination controversies, history is frequently used as a source of arguments (*8*,*9*), but the historical argument often is not based on up-to-date historical understanding. The polarizing controversies surrounding vaccination have never completely gone away, and the nearly unbroken tradition of debate apparently entices participants to reuse old arguments without making certain that their context is still valid. Vaccination involves national and international politics and the deeply personal sphere of child care. It is thus probably inevitable that culturally influenced ideas of bodily integrity and health from time to time are at odds with so-called vaccination technocracies (*10*).

## Alfred Russel Wallace

Alfred Russel Wallace’s humble origins contrast sharply with Charles Darwin’s privileged background. Wallace was born on January 8, 1823, in the Welsh village of Llanbadoc into an impoverished middle-class family. In 1836, when his parents could no longer support him, he was taken out of school to earn a living. He joined his brother John in London to work as a builder. In London, he regularly attended meetings at the Hall of Science in Tottenham Court Road, where followers of the utopian socialist Robert Owen lectured. Thus, as an adolescent, he became acquainted with radical sciences such as phrenology (*11*). In 1841, when Wallace was working as a land surveyor in Wales, a slump in business enabled him to devote more time to his developing interests in natural history. A few years later, while working as a teacher in Leicester, Wallace met the 19-year-old amateur entomologist Henry Walter Bates, who introduced him to beetle collecting. Wallace returned to Wales, but he stayed in touch with Bates; in their letters they discussed natural history and recent books. In 1847, inspired by reading the best-selling and scandalous Vestiges of the History of Creation, an anonymously published book that offered a naturalistic, developmental history of the cosmos and life, Wallace and Bates decided to travel to the Amazon River basin to study the origin of species, paying for their journey by working as professional specimen collectors.

Wallace spent the next 14 years of his life, interrupted only by a stay in England from October 1852 until early April 1854, collecting specimens in the Amazon Basin and the Malay Archipelago. As with Darwin, the geographic variation of supposedly stable species nurtured in Wallace the idea of organic change. An 1855 paper, On the Law Which Has Regulated the Introduction of New Species, is Wallace’s first formal statement of his understanding of the process of biological evolution. In this paper, he derives the law that “every species has come into existence coincident both in time and space with pre-existing closely allied species.” In February 1858, while having a severe malaria attack, Wallace connected the ideas of Thomas Malthus (1766–1834) on the regulation of populations with his earlier reasoning and developed a concept that was similar to Darwin’s mechanism of natural selection. Eager to share his discovery, Wallace wrote an essay on the subject as soon as he had recovered and sent it off to Darwin. This innocent act by Wallace set off the well-known and often recounted story of Darwin’s hurried writing and publication of On the Origin of Species.

Wallace returned to England in 1862 after the initial storm of reaction to Darwin’s theory had blown over. Together with Thomas Henry Huxley (1825–1895), he became one of the most vocal defenders of the theory of evolution. In the years up to 1880 he also wrote a large number of essays, letters, reviews and monographs that secured his position as one of the foremost naturalists in the United Kingdom; this status, however, did not translate into a permanent position or even some semblance of financial security. Only in 1881, after an intervention by Darwin and other eminent scientists, did he receive a Civil List Pension of 200£ per year. After 1880, having finished most of his major monographs, Wallace more and more directed his attention toward social issues and turned into a social radical—his conversion to spiritualism had already occurred in the 1860s. He remained faithful to his radical course until his death in 1913.

The first Vaccination Act in England was passed in 1840; it outlawed variolation (i.e., the practice of infecting a person with actual smallpox) and provided vaccination that used vaccines developed from cow pox or vaccinia virus free of charge. The 1853 Act made vaccination mandatory and included measures to punish parents or guardians who failed to comply. Changes in the law passed in 1867 permitted the authorities to enforce vaccination more efficiently. The law allowed the repeated prosecution of parents who failed to have their child vaccinated. The 1871 Act authorized the appointment of vaccination officers, whose task it was to identify cases of noncompliance. In 1889, in response to widespread public resistance, Parliament appointed a Royal Commission to draft recommendations to reform the system. The Commission published its conclusions in 1896. It suggested allowing conscientious objection, an exemption which passed into law in 1898. In the early 20th century, <200,000 exemptions were granted annually, representing ≈25% of all births (*12*).

The first vaccination act mainly incited resistance from heterodox medical practitioners who were forced out of business. Large-scale popular resistance began after the 1867 Act with its threat of coercive cumulative penalties. The social and political diversity of the British antivaccination movement is vividly described by Durbach (*12*). Many of the ≈200 organizations were quite eccentric, even by the standards of the time. However, Durbach’s analysis and other analyses (*13*) show that it is not correct to portray antivaccinationists indiscriminately as antirational, antimodern, and antiscientific. Just considering the details of the vaccination practice of the mid-19th century does much to make many criticisms understandable. For instance, the widespread arm-to-arm vaccination, used until 1898, carried substantial risks, and the instruments used (*14*) could contribute to severe adverse reactions. Furthermore, many antivaccinationists appealed, like their opponents, to enlightenment values and expertly used quantitative arguments.

Wallace himself apparently did not hold strong opinions about vaccination until the mid-1880s. He had received a vaccination as a young man before he left for South America, and all 3 of his children were vaccinated as well. Wallace was recruited some time in 1884 to the antivaccination movement through the efforts of his fellow spiritualist William Tebb (1830–1917), a radical liberal who in 1880 had cofounded the London Society for the Abolition of Compulsory Vaccination. Wallace’s commitment to the antivaccination cause was without doubt motivated by his social reformism, which in turn was underpinned by spiritualism and Swedenborgianism (*3*,*15*). These metaphysical foundations led him to a holistic view of health; he was convinced that smallpox was a contagious disease, but he also was certain that differences in susceptibility caused by nutritional or sanitary deficiencies played a major role in the epidemiology of the disease.

Despite his strong metaphysical commitments, Wallace, however, always remained a devoted empiricist and was among the first to use a statistics-based critique of a public health problem. Some of the groundwork for Wallace’s quantitative critique was laid by the highly regarded, but controversial, physicians Charles Creighton (1847–1927) and Edgar Crookshank (1858–1928). They attacked simplistic interpretations of and conclusions from Edward Jenner’s work (*16*) and demonstrated how difficult it is to determine vaccination success and vaccination status and to know what kind of contagion was actually used in an inoculation or vaccination. In works such as Vaccination Proved Useless and Dangerous (1889) or Vaccination a Delusion, Its Penal Enforcement a Crime (1898), Wallace mounted his attack on several claims: 1) that death from smallpox was lower for vaccinated than for unvaccinated populations; 2), that the attack rate was lower for vaccinated populations: and 3) that vaccination alleviates the clinical symptoms of smallpox.

Both provaccinationists and antivaccinationists relied heavily on time series of smallpox mortality rate data, which showed a general decline over the 19th century overlaid by several smaller epidemic peaks and the large pandemic peak of 1870–1873. Their conclusions from these data differed according to the way these data were subdivided into periods (*17*). For example, if it were assumed that vaccination rates increased in 1867, when cumulative penalties were introduced and fewer dared to challenge the vaccination law, and not in 1871, when the smallpox pandemic accelerated, then the rate of decline of smallpox mortality rates was lower when vaccination was more prevalent. Wallace concluded from his analysis that smallpox mortality rates increased with vaccination coverage, whereas his opponents concluded the exact opposite. Wallace argued that the problem of determining vaccination status was serious and undermined the claims of his opponents. He asserted that the physicians’ belief in the efficacy of vaccination led to a bias in categorizing persons on the basis of interpretation of true or false vaccination scars. Additionally, epidemiologic data for vaccination status were seriously incomplete. Depending on the sample, the vaccination status of 30%–70% of the persons recorded as dying from smallpox was unknown. Furthermore, if a person contracted the disease shortly after a vaccination, it was often entirely unclear if the patient should be categorized as vaccinated or unvaccinated. Provaccinationists argued that the error introduced by this ambiguity was most likely to be random and thus would not affect the estimate of the efficiency of the vaccine. In contrast, Wallace believed that doctors would have been more willing to report a death from smallpox in an unvaccinated patient and that this led to a serious bias and an overestimation of vaccine efficiency.

Wallace’s holistic conception of health influenced his argument as well. He was convinced that susceptibility to the disease of smallpox was not distributed equally across social classes. Weakened, poor persons living in squalor were in his opinion less likely to get vaccinated. At the same time they would have higher smallpox mortality rates because their living conditions made them more susceptible to the disease. He supported his hypothesis that susceptibilities differ with the observation that the mortality rate of unvaccinated persons had increased to 30% after the introduction of vaccination, while the vaccinated had enjoyed a slight survival advantage. This demonstrated to Wallace that factors other than vaccination must have played a major role.

## Conclusions

The numerical arguments used by Wallace and his opponents were based on an actuarial type of statistics, i.e., the analysis of life tables and mortalities. Inferential statistics that could be more helpful in identifying potential causes did not yet exist. The statistical approach to the vaccination debate used by Wallace and his opponents could simply not resolve the issue of vaccine efficiency; thus, each side was free to choose the interpretation that suited its needs best. However, despite its indecisive outcome, the debate was a major step in defining what kind of evidence was needed (*17*). It is also unjustified to portray the debate as a controversy of science versus antiscience because the boundaries between orthodox and heterodox science we are certain of today were far less apparent in the Victorian era (*18*). What the scope and methods of science were or should be were topics still to be settled. It is thus unwarranted to portray the 19th-century antivaccination campaigners generally as blindly religious, misguided, or irrational cranks. This judgment certainly does not apply to Alfred Russel Wallace.

Wallace was modern, but he represented an alternative version of modernity, a version that has been sidelined in historiography until recently but has lately been acknowledged as a central cultural feature of the late 19th century (*19*). Movements such as spiritualism were not resurrections of ancient traditions but used interpretations of the most recent natural science, such as experimental psychology, evolutionary biology, and astronomy (*20*), or electromagnetism (*21*). Some, like Wallace, also contested the social role that emerging professional sciences should play. Wallace strongly favored a natural science that also addressed moral, political, social, and metaphysical concerns, and with this inclination he ran against the tide that was more concerned with developing a barrier between politics and disinterested, objective science. In the case of vaccination, Wallace argued that liberty and science need to be taken into account, but that liberty is far more important than science. Wallace only appears to have been such a heretical figure if a large portion of the social, political, and intellectual reality of Victorian and Edwardian England is blotted out of the picture.

To argue that, then as now, the controversies are between religiously motivated, irrational eccentrics and rational, disinterested science is historically inaccurate and distracts from substantial differences in social, political, and economic context between then and now. The Victorian vaccination legislation was part of an unfair, thoroughly class-based, coercive, and disciplinary healthcare and justice system: poor, working-class persons were subjected to the full force of the law while better-off persons were provided with safer vaccines and could easily avoid punishment if they did not comply. The National Health Service, established in 1948, was planned to bring more social justice to health care. The new health system no longer was stigmatizing and coercive. The development has not stopped there: today, there is an increasingly strong emphasis on individual choice and involvement in decision making in the healthcare system in Great Britain. Patients have become customers. The contemporary vaccination controversy has to be seen against the opportunities and challenges offered within this new environment. It has become evident that population-based risk assessments of vaccine safety often fail to convince in this new context (*10*). Parents instead evince a clinical, individual-based attitude when assessing the risks of vaccination—their own children are often judged not to be average.

In Great Britain, such attitudes are reinforced by the recent developments, mentioned above, in the healthcare system that encourage choice and autonomy and also by individualized perspectives concerning parenting and child development. Such a clinical perspective of parents can, however, cut both ways. The individually witnessed causal relationship between therapy and recovery in the case of tetanus and diphtheria was instrumental in the widespread public acceptance of immunization (*17*). A similar mechanism is at play in the contemporary controversies: perceived causal relationships between vaccination and the appearance of complications undermine the claims that vaccines are generally safe.

This analysis also illustrates that contemporary vaccination controversies take place in specific historical contexts. Colgrove (*22*) depicts in detail how vaccination became an accepted public health intervention in the United States and what factors have fueled and influenced historical and contemporary controversies. For example, compared with most countries in Europe, the risk of costly litigation for pharmaceutical companies in the United States is much higher and the role of the state is seen as far more restricted. This specific background influences forms of provaccination and antivaccination campaigning, but it also needs to be taken into account that the increasing availability of Internet resources accessible from everywhere may contribute to making the arguments and the debate more uniform across the globe.

Modern vaccines save lives. But worries surrounding vaccination need to be taken seriously. And the lessons taught by history are, as usual, complex. As pointed out forcefully by Leach and Fairhead (*10*), vaccine delivery systems must suit social, cultural, and political realities. Paternalistic and coercive attitudes were harmful in the 19th century and are even less appropriate in the 21st century.
